# Influences of Separator Thickness and Surface Coating on Lithium Dendrite Growth: A Phase-Field Study

**DOI:** 10.3390/ma15227912

**Published:** 2022-11-09

**Authors:** Yajie Li, Liting Sha, Peili Lv, Na Qiu, Wei Zhao, Bin Chen, Pu Hu, Geng Zhang

**Affiliations:** 1 School of Materials Science and Engineering, Shanghai University, Shanghai 200444, China; 2 Tencent Inc., Shanghai 200233, China; 3 Mechanical and Electrical Engineering College, Hainan University, Haikou 570228, China; 4 Hubei Key Laboratory of Plasma Chemistry and Advanced Materials, School of Materials Science and Engineering, Wuhan Institute of Technology, Wuhan 430205, China; 5 Physical Sciences and Engineering Division, King Abdullah University of Science and Technology, Thuwal 23955-6900, Saudi Arabia

**Keywords:** lithium dendrite, phase-field simulation, separator, thickness, surface coating

## Abstract

Li dendrite growth, which causes potential internal short circuit and reduces battery cycle life, is the main hazard to lithium metal batteries. Separators have the potential to suppress dendrite growth by regulating Li^+^ distribution without increasing battery weight significantly. However, the underlying mechanism is still not fully understood. In this paper, we apply an electrochemical phase-field model to investigate the influences of separator thickness and surface coating on dendrite growth. It is found that dendrite growth under thicker separators is relatively uniform and the average dendrite length is shorter since the ion concentration within thicker separators is more uniform. Moreover, compared to single layer separators, the electrodeposition morphology under particle-coated separators is smoother since the particles can effectively regulate Li ionic flux and homogenize Li deposition. This study provides significant guidance for designing separators that inhibit dendrites effectively.

## 1. Introduction

Lithium metal is an ideal anode material for rechargeable batteries due to its high theoretical energy density and low redox potential. While the formation of lithium dendrites, which may penetrate the separator, causes short circuits and even thermal runaway; and it remarkably hinders the practical applications of lithium metal batteries [1]. The uncontrollable growth of lithium dendrites, coupled with the transport of Li^+^ and electrons as well as the electrochemical reactions at the anode-electrolyte interface, is an extremely complicated problem [2,3]. Many efforts have been devoted to handling this challenging problem, such as building 3D structure Li anodes [4,5], introducing solid electrolytes [6,7], functional separators [8,9], electrolyte additives [10,11], etc. Among them, functional separators are highly attractive since they not only act as physical barriers between electrodes but also effectively suppress dendrites by regulating Li^+^ ion distribution without increasing the weight of batteries conspicuously [12,13]. Considerable experimental studies have been conducted to explore the relation between Li deposition and separator structure [14,15]. For example, Ma et al. extracted eggshell membrane and investigated its performance as separator. Their results indicate that well-distributed pores and nanoporous structures allowing fast ion-diffusion can effectively suppress formation of Li dendrites even after long-term cycling at a high rate [16]. Shin et al. reported a thin nitrogen and sulfur codoped graphene (NSG) nanosheets coated polyethylene (PE) separator, which can effectively suppress dendrites due to the superior mechanical strength of NSG and the enhanced interfacial interaction between the NSG layer and Li metal [17].

In spite of massive screening experiments for designing separators with dendrite suppression, inhomogeneous Li^+^ flux and ion-concentration evolution can hardly be directly observed by experiments; thus, the mechanism of Li dendrite growth under different separators is still not fully understood. The phase-field method is a powerful computational approach for simulations of microstructural evolution of materials [18], which has been widely used in dynamic processes including solidification, solid-state phase transformation, grain growth, and electrochemical deposition [19]. In our previous review, we introduced the theoretical framework of the electrochemical phase-field model and reviewed existing phase-field simulations, especially for electrochemical dendrites [18]. For the application of phase-field model in dendrite growth under separators, Jana et al. explored the effects of pore size on Li dendrite growth through phase-field simulations. Four distinct regimes of dendrite growth were identified, specifically suppression, permeable, penetration, and short circuit regimes, which can serve as a guideline for designing improved separators [20]. Li et al. applied the phase-field model, which takes the separator phase into account to construct the total free energy, to study the ion re-distribution behavior and dendrite inhibition effects of porous separators with different pore sizes [21]. Nevertheless, the influence of separator thickness and surface coating on dendrite growth has not been fully understood. Herein, we apply the phase-field model to investigate the influences of separator thickness and surface coating on dendrite morphology, aiming to provide rational guidance for designing high safety battery separators.

## 2. Methods

The investigated system was a typical half-cell consisting of the lithium metal anode, electrolyte, and porous separator. The electrochemical phase-field model, proposed by Chen et al. [22], was used to investigate the effects of separator thickness and surface coating on ionic transport in the electrolyte and deposition on the electrode. We introduced two phase-field variables, ξ and ϕ (ranging from 0 to 1), and a concentration set, $\left\{c_{i}\right\}$ (i= Li, Li^+^, and anion $A^{m-}$), to describe the system. (ξ=1,ϕ=0) denotes the electrode, (ξ=0,ϕ=1) denotes the separator, and (ξ=0,ϕ=0) denotes the electrolyte. The total free energy of the system can be written as

(1) $$F=\int_{V}[f_{ch}(\xi ,\phi ,\{c_{i}\})+f_{grad}(\nabla \xi ,\nabla \phi )+f_{elec}(\{c_{i}\},\Phi )+f_{ns}(\xi )]dV$$

where Φ is the electric potential; $f_{ch}$, $f_{grad}$, and $f_{elec}$ are the chemical energy density, gradient energy density, and electrostatic energy density, respectively; $f_{ns}=h^{\prime }(\xi )\chi \psi$ is a noise function representing fluctuation, with an interpolating function $h^{\prime }(\xi )$, a random function χ, and the amplitude of fluctuation ψ.

The chemical free energy density is expressed as follows:

(2) $$\begin{array}{ll} f_{ch}(\xi ,\phi ,\{c_{i}\})= & \sum_{i}c_{i}\mu_{i}^{\circ }+RT\left(c_{{\mathrm{Li}}^{+}}\ln\frac{c_{{\mathrm{Li}}^{+}}}{c_{0}}+c_{A^{m-}}\ln\frac{c_{A^{m-}}}{c_{0}}\right)\\ & +W_{01}\xi^{2}{\left(1-\xi \right)}^{2}+W_{02}\phi^{2}{\left(1-\phi \right)}^{2}+W_{12}\xi^{2}\phi^{2} \end{array}$$

where $\mu_{i}^{\circ }$ is the reference chemical potential of component i; R is the molar gas constant; T is the temperature; and $c_{0}$ is the initial concentration of the electrolyte. The last three terms on the right side represent interphase barrier potentials, with constants $W_{01}$, $W_{02}$, and $W_{12}$. The gradient energy density is expressed as

(3) $$f_{grad}(\nabla \xi ,\nabla \phi )=\frac{1}{2}\kappa_{1}[1+\delta \cos(\omega \theta )]{\left(\nabla \xi \right)}^{2}+\frac{1}{2}\kappa_{2}{\left(\nabla \phi \right)}^{2}$$

where $\kappa_{1}$ and $\kappa_{2}$ are the gradient energy coefficients; δ is the anisotropic strength; ω is the anisotropic mode; θ is the angle between the interface normal vector and the reference axis. The electrostatic energy density is expressed as

(4) $$f_{elec}(\left\{c_{i}\right\},\Phi )=\sum_{i}z_{i}Fc_{i}\Phi$$

where F is the Faraday constant and $z_{i}$ is the valence of component *i*.

The electrochemical reaction rate can be expressed from the Butler–Volmer equation. Thus, the evolution equations for ξ and ϕ are

(5) $$\frac{\partial \xi }{\partial t}=-L_{1}\frac{\delta f}{\delta \xi }-R_{\eta }h^{\prime }(\xi )\left(e^{\frac{(1-\alpha )F\eta }{RT}}-\frac{c_{{\mathrm{Li}}^{+}}}{c_{0}}e^{\frac{-aF\eta }{RT}}\right)$$

(6) $$\frac{\partial \phi }{\partial t}=-L_{2}\frac{\delta f}{\delta \phi }$$

where $L_{1}$ and $L_{2}$ are the interfacial mobilities of the electrode and separator, respectively; $R_{\eta }$ is the reaction constant; α and 1−α are the charge-transfer coefficients; and $h(\xi )=\xi^{3}(6\xi^{2}-15\xi +10)$ is an interpolating function. The overpotential is defined as $\eta =\Phi -\Phi_{0}$, where Φ is the potential of the Li metal anode and $\Phi_{0}$ is the equilibrium potential without electric current. The evolution equation of Li^+^ concentration is

(7) $$\frac{\partial c_{{\mathrm{Li}}^{+}}}{\partial t}=\nabla \cdot \left(D_{{\mathrm{Li}}^{+}}\nabla c_{Li^{+}}+D_{{\mathrm{Li}}^{+}}c_{{\mathrm{Li}}^{+}}\frac{F}{RT}\nabla \Phi \right)-K\frac{\partial \xi }{\partial t}$$

where $D_{{\mathrm{Li}}^{+}}$ is the diffusion coefficient of Li^+^ and K is the accumulation constant. The evolution equation of electrostatic potential is described by the law of charge conservation

(8) $$\nabla \cdot (\sigma_{\mathrm{eff}}\nabla \Phi )=Fc_{\mathrm{Li}}\frac{\partial \xi }{\partial t},$$

where $\sigma_{\mathrm{eff}}=h(\xi )\sigma_{\mathrm{Li}}+[1-h(\xi )]\sigma_{e}$ is the effective electric conductivity and $\sigma_{\mathrm{Li}}$ and $\sigma_{e}$ are the electric conductivity of electrode and electrolyte, respectively.

All simulations in this work were performed with COMSOL Multiphysics 5.6 using the finite element method. The phase-field simulations of the Li plating morphology were conducted in a two-dimensional half-cell electrodeposition system with a size of 8 × 8 μm^2^. The simulation domain was discretized by quadrilateral mesh at a minimum size of 0.0005 μm and a maximum size of 0.5 μm. Adaptive mesh refinement was used for improving the convergence and accuracy of the simulations. The adiabatic boundary condition was used for the four boundaries of the phase-field variable and for the left and right boundaries of the concentration and electric potential. The top and bottom boundaries of Li ion concentration were set as 1 M and 0 M, while the electric potential at the top and bottom boundaries were set as 0 V and −0.1 V. Detailed parameters are listed in Table 1.

The simulations presented in this work focus on the dendrite inhibition of separator thickness and surface coating. Accordingly, a number of simplifications were made, e.g., assuming a concentration-independent diffusivity and neglecting the mechanical dendrite-separator interactions, electro-convection effect, and secondary reactions. Separators with idealized pores were utilized to investigate the effects of separator thickness and surface coating on ionic transport. These idealized pore separators allowed different characteristics to be decoupled.

## 3. Results and Discussion

### 3.1. Li Dendrite Growth in Batteries without Separators

**Figure 1:**
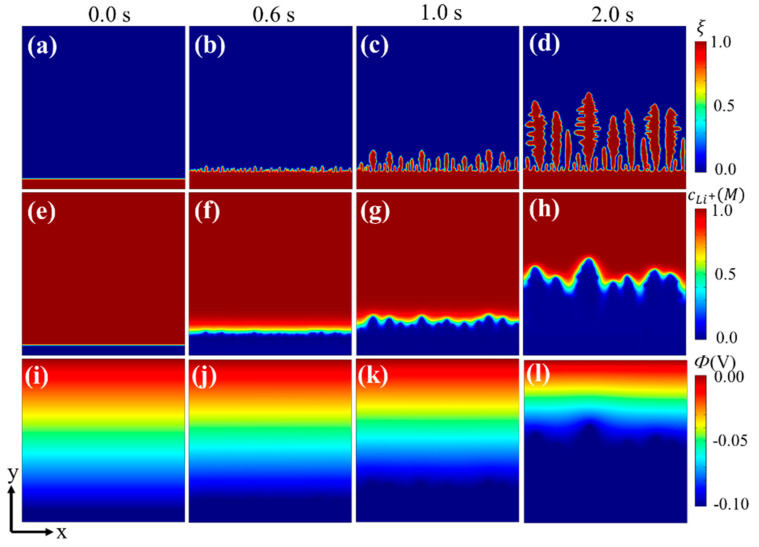
Phase-field simulation for Li dendrite growth without separator: (**a**–**d**) phase-field variable, (**e**–**h**) Li^+^ concentration, and (**i**–**l**) electric potential.

First, dendrite growth without a separator was conducted to test the validity of the electrochemical phase-field model. In this study, we introduced a noise function $f_{ns}(\xi )$ to simulate the local thermal and structural heterogeneity stemming from anodic defects, imperfect contact with the interface, local temperature variations, etc. [23] Electrodeposition is an intrinsic process without initial artificial nuclei, which can avoid disturbances resulting from the size, shape, and orientation of artificial nuclei. The initial phase-field variable (ξ), Li^+^ concentration ($c_{{\mathrm{Li}}^{+}}$), and electric potential (Φ) are shown in Figure 1a,e,i. The electrolyte has a high Li^+^ concentration (ξ=0) and the anode has zero Li^+^ concentration (ξ=1). The electric potential of the anode was fixed at −0.1 V, which linearly increases from the electrode/electrolyte interface to the top of the simulation area (Figure 1i). The foregoing parameters remain the same for all simulations hereafter unless specified otherwise. A large concentration and electric potential gradient occurred near the anode/electrolyte interface, which acts as the driving force of Li electrodeposition.

During the charging process, the evolution of the phase-field variable, the Li^+^ concentration, and the electric potential are shown in Figure 1. After an electrodeposition process of 0.6 s (Figure 1b,f,j), the roughness of the interface occurred obviously. With formation of small nuclei at the interface, the tip region had a slightly higher concentration than the valley region. Further growth of these nuclei led to the formation of long dendrite-like patterns (Figure 1c,d). The difference between the Li^+^ concentration surrounding the dendrite tips and valley regions continued to grow (Figure 1g,h), since the Li^+^ diffusion paths of dendrite tips were shorter than that of the valley regions [23]. This distribution further promoted Li deposition on the dendrite tips. With dendrite growth, the electric potential near the dendrite tip was lower than the surrounding valley region (Figure 1l); thus, there appeared a transverse component for the electric field, which facilitates Li^+^ transport from both the electrolyte and valley regions to the dendrite tips and further promotes dendrite growth [23]. The simulation results are in good agreement with experiments and thus show the validity of the electrochemical phase-field model above.

### 3.2. Effects of Separator Thickness on Li Dendrite Growth

**Figure 2:**
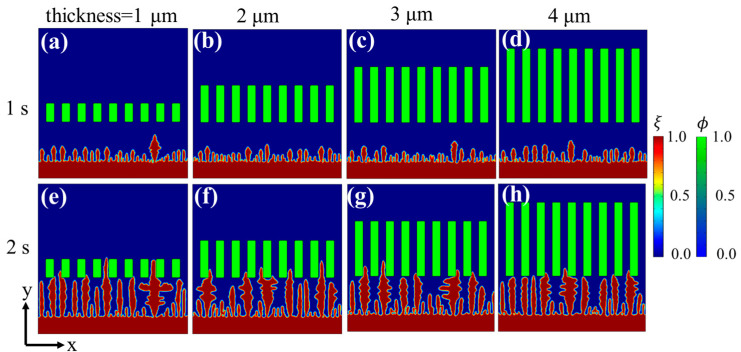
Phase-field simulation of Li deposition under separators with different thicknesses: (**a**–**d**) phase-field variable at 1 s, (**e**–**h**) phase-field variable at 2 s.

In order to investigate the effect of separator thickness on the Li deposition, the simulation above was further extended to the case with a separator. The effect of separator thickness on dendrite growth was investigated by conducting phase-field simulations with four kinds of porous separators (thicknesses: 1 μm, 2 μm, 3 μm, and 4 μm) (Figure 2). The results indicated that with the increase of separator thickness, electrodeposition becomes more homogeneous and the average height is shorter.

**Figure 3:**
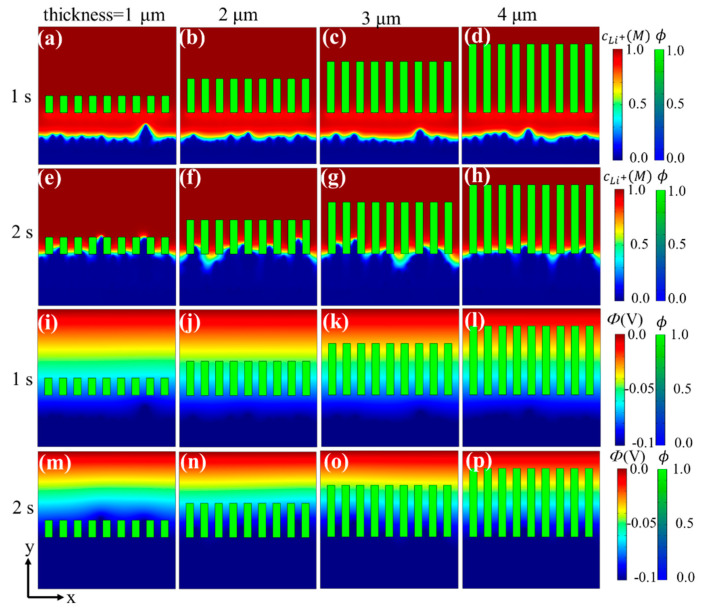
Phase-field simulation of Li deposition under separators with different thicknesses: (**a**–**d**) Li^+^ distribution at 1 s, (**e**–**h**) Li^+^ distribution at 2 s, (**i**–**l**) electric potential at 1 s, (**m**–**p**) electric potential at 2 s.

The evolution of the Li^+^ concentration and the electric potential in the above four cases at 1 s and 2 s are shown in Figure 3. It can be observed that during the nucleation and initial stages of dendrite growth (Figure 3a–d,i–l), both the lithium ion and electric potential distributions were similar under different separators. While electrodeposition occurred, the lithium ion and electric potential distributions under different separators were quite different (Figure 3e–h,m–p). After a period of initial deposition, there were several dendrite tips under each separator, which produces a transverse electric field and causes “ion tip aggregation effect” that accelerates uneven dendrite growth. For thicker separators, the lithium ions can be divided and constrained within longer channels, thus retarding the transverse migration of lithium ions, eventually generating a more uniform Li^+^ flux/electric field distribution, and, therefore, yielding a more homogenous Li deposition. In contrast, the ion distribution under thinner separators is heterogeneous and causes a larger concentration/electric potential gradient near the dendrite tips, and, accordingly, aggravates uneven dendrite growth. Consequently, the design and fabrication of thicker separators is needed to promote more uniform mass transport and suppress dendrite growth.

### 3.3. Effects of Surface Coating on Li Dendrite Growth

In recent years, separators with various surface modifications for Li dendrite suppression have been reported, while most of them mainly focus on preventing dendrite proliferation and growth, including enhancing the mechanical strength of separators and dissolving dendrites with Li reactions [14,24]. However, the essentially inhomogeneous Li^+^ flux that leads to dendrite nucleation and growth has not been addressed, thus limiting the improvement of electrochemical performance.

**Figure 4:**
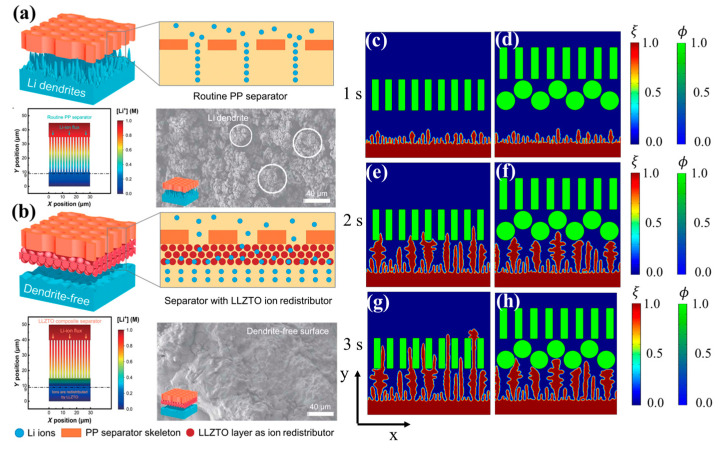
Schematics, ionic distribution, and SEM images of Li metal anodes using (**a**) PP separator and (**b**) LLZTO coated PP separator [25]; simulation results of phase-field variable under (**c**,**e**,**g**) single layer separator and (**d**,**f**,**h**) coated separator.

Li^+^ can only migrate through the separator pores in routine Li metal batteries; therefore, Li^+^ are very crowded near the pores after crossing the separator. This leads to the enrichment of Li^+^ on the anode around the separator pores and the impoverishment of Li^+^ around the separator skeletons (Figure 4a). Zhang et al. [25] reported on a composite separator consisting of a Li_6.75_La_3_Zr_1.75_Ta_0.25_O_12_ (LLZTO) coated polypropylene (PP) separator (Figure 4a), which has abundant three-dimensional ion-conduction channels to release ionic congestion and guide Li^+^ to deposit uniformly (Figure 4b). Herein, we will further explore the effects of surface coating on Li deposition through phase-field simulations. Figure 4c–h exhibit Li deposition during 1–3 s under a single layer separator and coated separator. For the single layer separator, Li dendrites grew quickly without hindrance from protective layers. On the other hand, the separator with a particle coating reduced the formation of long and uneven dendrites. The tips of sharp dendrites were deflected once they contacted the curved surface of the particle coating. The tortuosity generated by curved and narrow channels between particles increases the length of pathways for Li dendrites, thus protecting the separator from puncturing.

**Figure 5:**
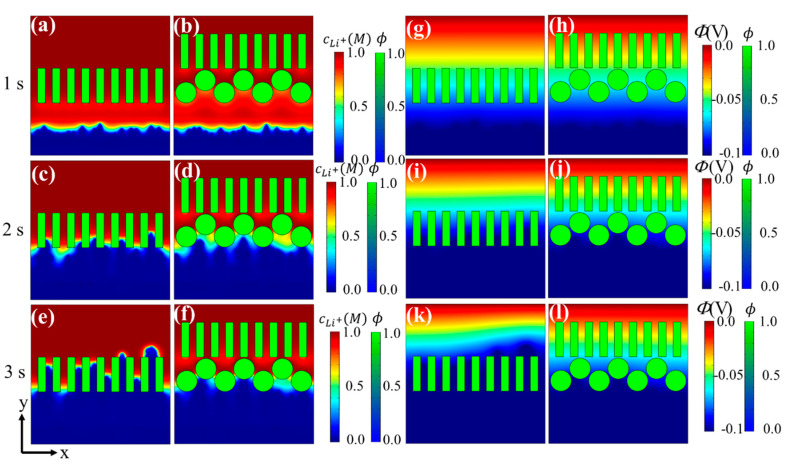
Li^+^ concentration under (**a**,**c**,**e**) single layer separator and (**b**,**d**,**f**) coated separator, and electric potential under (**g**,**i**,**k**) single layer separator and (**h**,**j**,**l**) coated separator.

The distributions of lithium ions and electric potential under the above two cases at 1 s, 2 s, and 3 s are shown in Figure 5. Normally, Li^+^ moves more slowly than electrons, so concentration polarization occurs. At the early stage, concentration polarization under the coated separator was slightly more severe than that under the single layer separator since the coated round particles cause slightly higher ion concentration fluctuation. When dendrites approached the separator, the particle coating reduced the growth speed of the dendrites and Li deposition became more uniform because: (1) the particles and the inter-particle channels can regulate movement of Li^+^ and homogenize its distribution; and (2) coated round particles can change dendrite growth direction, promote lateral growth, decrease dendrite growth speed, and smooth the deposition morphology, which in turn homogenizes lithium ions and the electric potential distribution.

## 4. Conclusions

We used the electrochemical phase-field model to simulate the effects of separator thickness and surface coating on Li dendrite morphology. Compared with thinner separators, dendrite growth under thicker separators was relatively uniform and the average dendrite length was shorter since the ion concentration within thicker separators was more uniform. The surface coating of separators can reduce the dendrite growth speed by deflecting their tips and prolonging their growth pathways. Moreover, surface coating can regulate Li^+^ movement and homogenize Li^+^ distribution, thus suppressing Li dendrite growth. This work will motivate both theoretical and experimental studies on the effects of separators on Li dendrite growth in the future.

## Figures and Tables

**Table 1:** Parameters of the phase-field model.

Parameters	Symbol	Value
Interfacial mobility 1	$L_{1}$	$5\times 10^{-5}{\;m}^{3}\cdot J^{-1}\cdot s^{-1}$
Interfacial mobility 2	$L_{2}$	$1\times 10^{-6}{\;m}^{3}\cdot J^{-1}\cdot s^{-1}$
Reaction constant	$R_{\eta }$	$25{\;s}^{-1}$
Gradient energy coefficient 1	$\kappa_{1}$	$8.33\times 10^{-11}\;J\cdot m^{-1}$
Gradient energy coefficient 2	$\kappa_{2}$	$1.67\times 10^{-10}\;J\cdot m^{-1}$
Anisotropic strength	δ	0.05
Anisotropic mode number	ω	4
Barrier height 1	$W_{01}$	$6\times 10^{5}\;J\cdot m^{-3}$
Barrier height 2	$W_{02}$	$6\times 10^{5}\;J\cdot m^{-3}$
Barrier height 3	$W_{12}$	$6\times 10^{5}\;J\cdot m^{-3}$
Diffusion coefficient of Li^+^	$D_{{\mathrm{Li}}^{+}}$	$3\times 10^{-13}{\;m}^{2}\cdot s^{-1}$
Li^+^ concentration of electrolyte	$c_{0}$	$1\times 10^{3}\;\mathrm{mol}\cdot m^{-3}$
Charge transfer coefficient	α	0.5
Electric conductivity of electrode	$\sigma_{\mathrm{Li}}$	$1\times 10^{7}\;S\cdot m^{-1}$
Electric conductivity of electrolyte	$\sigma_{e}$	$0.1\;S\cdot m^{-1}$
Accumulation constant	K	$1.92\times 10^{3}\;\mathrm{mol}\cdot m^{-3}$
Temperature	T	298.15 K

## Data Availability

Data are contained within the article.

## References

[1] Wu F., Yuan Y.-X., Cheng X.-B., Bai Y., Li Y., Wu C., Zhang Q. (2018). Perspectives for restraining harsh lithium dendrite growth: Towards robust lithium metal anodes. Energy Storage Mater..

[2] Pang Q., Liang X., Shyamsunder A., Nazar L.F. (2017). An in vivo formed solid electrolyte surface layer enables stable plating of Li metal. Joule.

[3] Mendizabal A.O., Gomez N., Aguesse F., López-Aranguren P. (2021). Designing Spinel Li_4_Ti_5_O_12_ Electrode as Anode Material for Poly(ethylene) oxide-Based Solid-State Batteries. Materials.

[4] Zhang Y., Wang C., Pastel G., Kuang Y., Xie H., Li Y., Liu B., Luo W., Chen C., Hu L. (2018). 3D Wettable Framework for Dendrite-Free Alkali Metal Anodes. Adv. Energy Mater..

[5] Zheng Z.-J., Su Q., Zhang Q., Hu X.-C., Yin Y.-X., Wen R., Ye H., Wang Z.-B., Guo Y.-G. (2019). Low volume change composite lithium metal anodes. Nano Energy.

[6] Wang X., Zhai H., Qie B., Cheng Q., Li A., Borovilas J., Xu B., Shi C., Jin T., Liao X. (2019). Rechargeable solid-state lithium metal batteries with vertically aligned ceramic nanoparticle/polymer composite electrolyte. Nano Energy.

[7] Akkinepally B., Reddy I.N., Ko T.J., Yoo K., Shim J. (2022). Dopant effect on Li+ ion transport in NASICON-type solid electrolyte: Insights from molecular dynamics simulations and experiments. Ceram. Int..

[8] Lin D., Zhuo D., Liu Y., Cui Y. (2016). All-integrated bifunctional separator for Li dendrite detection via novel solution synthesis of a thermostable polyimide separator. J. Am. Chem. Soc..

[9] Chen M., Shao M., Jin J., Cui L., Tu H., Fu X. (2022). Configurational and structural design of separators toward shuttling-free and dendrite-free lithium-sulfur batteries: A review. Energy Storage Mater..

[10] Shi F., Pei A., Vailionis A., Xie J., Liu B., Zhao J., Gong Y., Cui Y. (2017). Strong texturing of lithium metal in batteries. Proc. Natl. Acad. Sci. USA.

[11] Sreedeep S., Natarajan S., Aravindan V. (2022). Recent advancements in LiCoPO_4_ cathodes using electrolyte additives. Curr. Opin. Electrochem..

[12] Ren W., Zheng Y., Cui Z., Tao Y., Li B., Wang W. (2021). Recent progress of functional separators in dendrite inhibition for lithium metal batteries. Energy Storage Mater..

[13] Ding F., Xu W., Graff G.L., Zhang J., Sushko M.L., Chen X., Shao Y., Engelhard M.H., Nie Z., Xiao J. (2013). Dendrite-free lithium deposition via self-healing electrostatic shield mechanism. J. Am. Chem. Soc..

[14] Zhang S.S., Fan X., Wang C. (2018). Preventing lithium dendrite-related electrical shorting in rechargeable batteries by coating separator with a Li-killing additive. J. Mater. Chem. A.

[15] Kim P.J.H., Pol V.G. (2019). Surface functionalization of a conventional polypropylene separator with an aluminum nitride layer toward ultrastable and high-rate lithium metal anodes. ACS Appl. Mater. Interfaces.

[16] Ma L., Chen R., Hu Y., Zhang W., Zhu G., Zhao P., Chen T., Wang C., Yan W., Wang Y. (2018). Nanoporous and lyophilic battery separator from regenerated eggshell membrane with effective suppression of dendritic lithium growth. Energy Storage Mater..

[17] Shin W.-K., Kannan A.G., Kim D.-W. (2015). Effective suppression of dendritic lithium growth using an ultrathin coating of nitrogen and sulfur codoped graphene nanosheets on polymer separator for lithium metal batteries. ACS Appl. Mater. Interfaces.

[18] Wang Q., Zhang G., Li Y., Hong Z., Wang D., Shi S. (2020). Application of phase-field method in rechargeable batteries. NPJ Comput. Mater..

[19] Chen L.-Q. (2002). Phase-field models for microstructure evolution. Annu. Rev. Mater. Res..

[20] Jana A., Ely D.R., García R.E. (2015). Dendrite-separator interactions in lithium-based batteries. J. Power Sources.

[21] Li Y., Zhang G., Chen B., Zhao W., Sha L., Wang D., Yu J., Shi S. (2022). Understanding the separator pore size inhibition effect on lithium dendrite via phase-field simulations. Chin. Chem. Lett..

[22] Chen L., Zhang H.W., Liang L.Y., Liu Z., Qi Y., Lu P., Chen J., Chen L.-Q. (2015). Modulation of dendritic patterns during electrodeposition: A nonlinear phase-field model. J. Power Sources.

[23] Hong Z., Viswanathan V. (2018). Phase-field simulations of lithium dendrite growth with open-source software. ACS Energy Lett..

[24] Yu B.-C., Park K., Jang J.-H., Goodenough J.B. (2016). Cellulose-based porous membrane for suppressing Li dendrite formation in lithium–sulfur battery. ACS Energy Lett..

[25] Zhao C.-Z., Chen P.-Y., Zhang R., Chen X., Li B.-Q., Zhang X.-Q., Cheng X.-B., Zhang Q. (2018). An ion redistributor for dendrite-free lithium metal anodes. Sci. Adv..

